# Traditional Chinese acupuncture versus minimal acupuncture for mild-to-moderate knee osteoarthritis: a protocol for a randomised, controlled pilot trial

**DOI:** 10.1136/bmjopen-2016-013830

**Published:** 2016-12-12

**Authors:** Ning Sun, Guang-Xia Shi, Jian-Feng Tu, Yong-Ting Li, Li-Wen Zhang, Yan Cao, Yi Du, Jing-Jie Zhao, Da-Chang Xiong, Hai-Kun Hou, Cun-Zhi Liu

**Affiliations:** 1 Department of Acupuncture and Moxibustion, Beijing Hospital of Traditional Chinese Medicine affiliated to Capital Medical University, Beijing, China; 2 Department of Medicine, School of Medicine, Shandong University of Traditional Chinese Medicine, Jinan, China; 3 Department of Traditional Chinese Medicine, Beijing Friendship Hospital, Capital Medical University, Beijing, China; 4 Department of Acupuncture and Moxibustion, Beijing Jishuitan Hospital, Peking University, Beijing, China

**Keywords:** Knee Osteoarthritis, Acupuncture, PAIN MANAGEMENT

## Abstract

**Introduction:**

Knee osteoarthritis (KOA) is one of the most common musculoskeletal disorders. Acupuncture is a popular form of complementary medicine for musculoskeletal conditions, although the evidence is inconclusive. Our objective is to evaluate the efficacy of traditional Chinese acupuncture for pain relief and function improvement in mild-to-moderate knee osteoarthritis (TCAKOA) participants.

**Methods/analysis:**

42 patients will be recruited who have been diagnosed with mild-to-moderate KOA and randomly allocated in equal proportions to traditional Chinese acupuncture or minimal acupuncture. They will receive acupuncture for 24 sessions over 8 weeks. The primary end point is success rate, which will be calculated according to a change from baseline in Western Ontario and McMaster Universities Osteoarthritis Index pain and function scores at 8 weeks. Secondary end points include pain and function measurement, global change, the quality of life and the use of non-steroidal anti-inflammatory drugs (Celebrex, Pfizer) at 8, 16 and 26 weeks.

**Ethics and dissemination:**

Ethical approval of this study has been granted by the Research Ethical Committee of Beijing Hospital of Traditional Chinese Medicine Affiliated to Capital Medical University (permission number: 2016BL-010-02). Written informed consent will be obtained from all participants. Outcomes of the trial will be disseminated through peer-reviewed publications.

**Trial registration number:**

ISRCTN14016893; Pre-results.

## Background

Knee osteoarthritis (KOA) is one of the most common musculoskeletal disorders,^1^ which features as a protracted course of disease. A systematic review shows that the prevalence of KOA is 27.3% in women and 21.0% in men.^2^ A cross-sectional study with 9512 participants aged 50 years or older shows that the prevalence of radiographic KOA was 43.8% in women and 21.1% in men in South Korea.^3^ KOA is one of the leading causes of pain and global disability.

The objective of treating KOA is the alleviation of pain and improving quality of life. Five guidelines^1^ ^4–6^ have evaluated treatment effects on key outcomes of KOA (including pain, function and disability). Pharmacologic agents, comprising non-opioid/opioid oral, non-steroidal anti-inflammatory drugs (NSAIDs) oral, intra-articular steroid, topical analgesics and hyaluronate injections are normally used but may be associated with significant adverse reactions (such as peptic ulcer, hypertension and renal damage).^6–8^ Guidelines emphasise the potential role of non-pharmacologic treatment, such as aerobic exercise, electrical nerve stimulation, acupuncture in the treatment. Effective alternatives to pharmacological are therefore desirable.

Traditional Chinese acupuncture (TCA) is a popular form of complementary medicine. In 2005, Germany, Witt and colleagues showed that 8 weeks of the semistandardised acupuncture treatment had significantly alleviated the patient's pain and dysfunction contrasted to the minimal acupuncture (MA) treatment and no treatment condition.^9^ A meta-analysis showed that acupuncture could be considered to be an effective physical treatment for KOA.^10^ However, in the October 2014 publication of *JAMA*, Dr Hinman *et al* conducted a Zelen design clinical trial to investigate acupuncture for patients suffering from chronic knee pain. The investigation declared that acupuncture did not convey more advantages compared to sham or better function in sufferers with mild or harsh chronic knee pain.^11^ However, flaws may exist in the trial design, statistics, interpretation of the results.^12–26^ First of all, participants aged ≥50 years with moderate-to-severe chronic knee pain have been recruited. These inclusion criteria may be more suitable for arthroscopic or joint replacement therapy according to the guidance.^1^ ^4^ Second, acupuncture intervention is 8–12 sessions in total. The dosage for acupuncture is far from sufficient.^14^ ^16^ Third, the team registered trial as studying laser acupuncture, instead of traditional acupuncture.^27^ Researchers had changed the main aim selectively.^14^ ^26^ At present, there is a controversy over whether the acupuncture has benefit for KOA.^1^ ^4^ Therefore, our aim is to investigate the intensive TCA for participants with mild-to-moderate KOA.

## Materials and methods

### Study design

**Figure 1:**
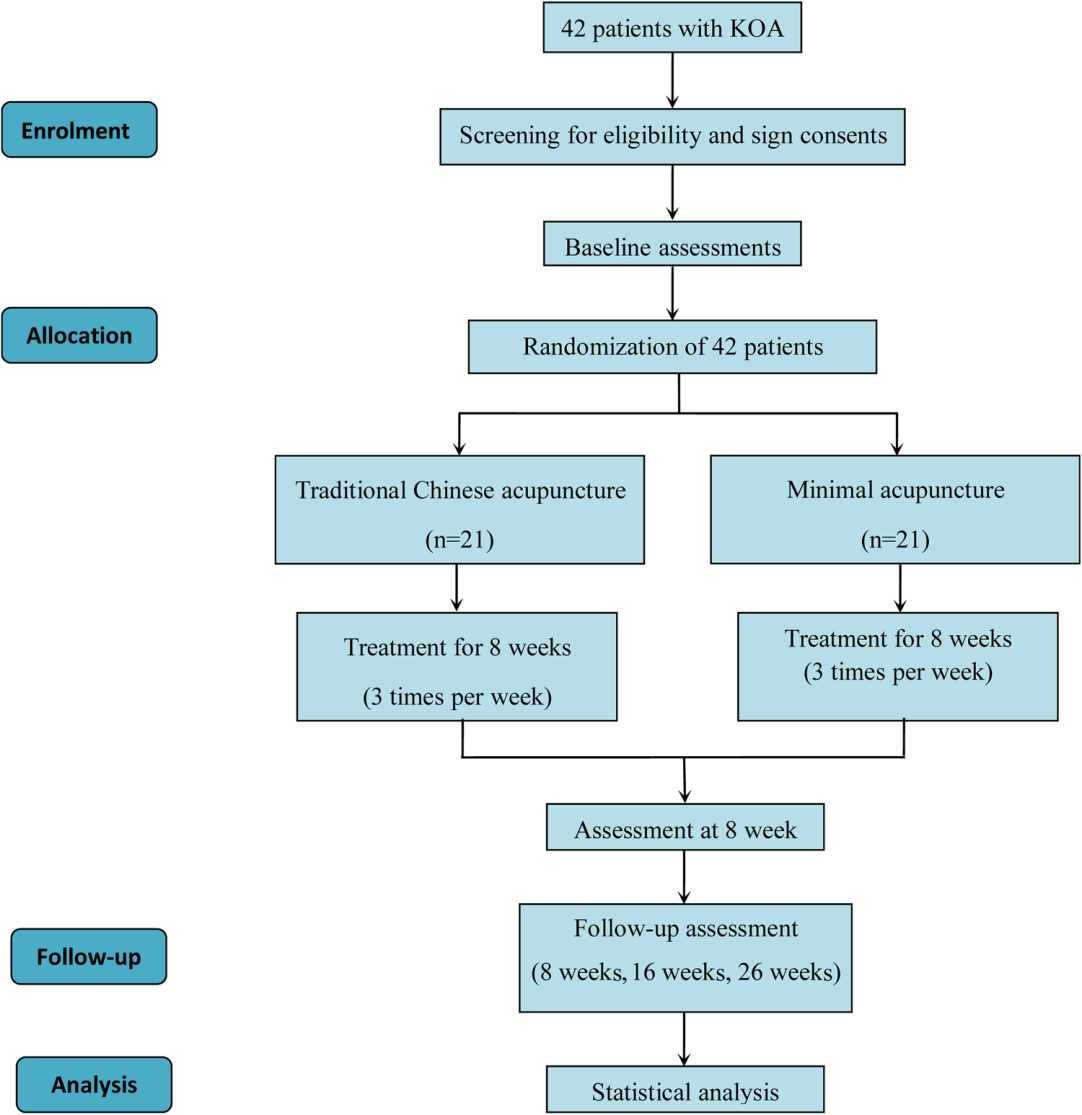
Trial flow chart. KOA, knee osteoarthritis.

The study proposes a two-arm, randomised, clinical pilot trial. We will enrol patients from Beijing Hospital of Traditional Chinese Medicine Affiliated to Capital Medical University, Beijing Friendship Hospital and Beijing Jishuitan Hospital. The trial has been registered with ISRCTN at Current Controlled Trials (ISRCTN14016893). Some recruitment strategies include radio and print advertisements through the local web sites and community centre as well as recruiters with general practitioners. The intervention includes 24 sessions of acupuncture and 3 times follow-up (figure 1 and table 1).

**Table 1:** Time to visit and data collection

	Baseline	Treatment phase		Follow-up phase	
	−1 day	0 day	8 weeks	16 weeks	26 weeks
Patients					
Informed consent	×				
Sign informed consent		×			
Medical history	×				
Physical examination	×				
Randomisation		×			
Intervention TCA group (n=21)		24 sessions of TCA			
Comparisons MA group (n=21)		24 sessions of MA			
Outcomes					
WOMAC		×	×	×	×
KOOS		×	×	×	×
VAS		×	×	×	×
SF-12		×	×	×	×
The use of NSAIDs			×	×	×
Participant safety					
Adverse events		×	×	×	×

KOOS, Knee injury and Osteoarthritis Outcome Score; NSAIDs, non-steroidal anti-inflammatory drugs; MA, minimal acupuncture; SF-12, 12-item Short Form Health Survey; TCA, traditional Chinese acupuncture; WOMAC, Western Ontario and McMaster Universities Osteoarthritis Index.

### Inclusion criteria

1. Age 45–75 years (either sex)
2. Chronic knee pain for the last 6 months
3. Morning stiffness ≤30 min.

The above-mentioned criteria are consistent with the National Institute for Health and Clinical Excellence (NICE) Guidelines 2014 Edition.^4^

1. Radiologic confirmation of KOA (Kellgren–Lawrence grade II or III 28).

### Exclusion criteria

1. Recent acupuncture
2. Other sickness impact the knee
3. On surgical operation list
4. Neurologic as well as psychiatric diseases
5. Severe coagulopathy
6. Breastfeeding or pregnancy
7. Not fitting to take the NSAID (Celebrex, Pfizer) provided.

For bilaterally eligible knees, the most symptomatic side will be evaluated in the course of the study.

### Randomisation and allocation concealment

Eligible patients will be randomly assigned to the TCA group or the MA group in a ratio of 1:1 through central automated allocation procedures. An independent statistician generates randomisation sequence by using the SAS V.9.1.3 statistical package (SAS Institute, Cary, North Carolina, USA). Acupuncturists will not involve in the process of randomisation.

The research assistants who collect data, the statisticians who assess outcomes and make statistical analysis will be blinded to group assignment. Participants will not be disclosed information regarding the allocation. Administrators will not be blinded because of the nature of intervention.

### Interventions

The acupuncture protocol follows the CONSORT^29^ and STRICTA.^30^ All acupuncturists have Chinese medicine practitioner licenses, and they have been qualified for at least 10 years. All acupuncturists will receive training in the application of MA. Celebrex will give to participants if their pain intensity ≥80 on a 10 cm visual analogue scale (VAS).^31^

The protocol specifies the intervention of acupuncture to be a 20 min treatment which is applied 3 times weekly for 8 weeks, with 24 sessions in total permitted. Disposable, sterile steel, 0.30×25 mm or 0.30×40 mm needles (Huatuo disposable acupuncture needle, Suzhou Medical Co., Jiangsu, China) will be used in two groups.

### TCA group

**Figure 2:**
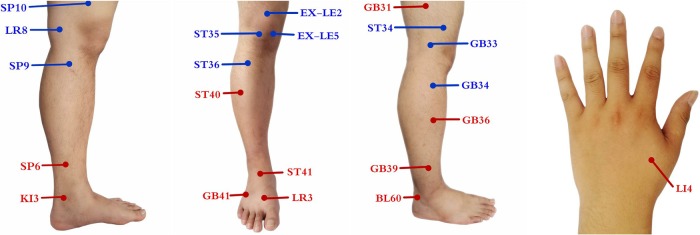
The points used in the TCA group. TCA, traditional Chinese acupuncture.

Acupuncture points are selected on traditional Chinese Medicine theory of the ‘Bi’ syndrome. These points are composed of 10 local points (*ST34, ST35, ST 36, EX-LE2, EX-LE5, GB33, GB34, SP9, SP10, LV8*) and 11 distal points (*GB31, GB36, GB39, GB41, ST40, ST41, LR3, BL60, SP6, KI3, LI4*) (figure 2). Physicians can choose five to six local points and three to four distal points. Needles will be making an optimum insertion into the skin. Acupuncturists are instructed to achieve ‘De Qi’, and needles will be stimulated manually at least 10 s.

### MA group

Non-acupoints in a superficial puncture (2 mm in depth) will be performed in the MA group. Treatment is standardised needling without manual stimulation at seven points at certain distances from TCA group points (table 2). The MA procedure will be given on the same schedule as the TCA group.

**Table 2:** Sham acupuncture points in the MA group

Sham acupuncture points	Location
MP1	Ulnar margin of forearm, midpoint of the connecting line between the rasceta head and condylus medialis humeri
MP2	2 cun above the malleolus lateralis, between the gall bladder meridian and stomach meridian on the distal part of the fibula
MP3	2 cun above the malleolus medialis, in the centre of the tibia surface area (intracutaneous without periost contact, in the direction towards the knee)
MP4	Midpoint of the connecting line between ST36 and GB34
MP5	6 cun above the upper edge of the patella (between the spleen and stomach meridian)
MP6	5 cun above the upper edge of the patella (between the spleen and stomach meridian)
MP7	4 cun above the upper edge of the patella (between the spleen and stomach meridian)
MP8	1 cun under the tibia head, in the medial edge of leg
MP9	Midpoint of the connecting line between GB40 and ST41
MP10	3 cun above the medial edge of calcaneal

One ‘cun’ is defined according to the rules of traditional Chinese medicine as the width of the interphalangeal joint of patient's thumb.

MA, minimal acupuncture.

## Outcomes

### Primary outcome measurement

Success rate will be calculated according to a change from baseline in Western Ontario and McMaster Universities Osteoarthritis Index (WOMAC)^32^ ^33^ pain and function scores at 8 weeks. WOMAC function subscale (17 items, scored from 0 to 68) and pain subscale (5 items, scored from 0 to 20) with higher scores represent worse pain and function.

### Secondary outcome measurement

Knee pain will be assessed by WOMAC pain subscale and Visual Analogue Scale (VAS, 0–100, higher scores representing worse pain). WOMAC function subscale will be used to measure physical function. Knee injury and Osteoarthritis Outcome Score (KOOS 0–100, higher scores indicating better function) subscales comprise pain, symptoms, activities of daily living and quality of life.^34^ Health-related quality of life will use the 12-item Short Form Health Survey (SF-12 0–100, higher scores representing better quality of life).^35^ The use of the NSAIDs (Celebrex, Pfizer) will be at 8, 16 and 26 weeks.

Adverse events will be monitored and reported by acupuncturists via open-ended questioning. Patients will be suggested to state any adverse circumstances they go through, comprising discomfort or bruise in the locations pierced by needle, nausea or the feeling of faint after the acupuncture treatment. Every crucial sign and adverse events are going to be investigated and recorded during every visit.

### Sample size

The purpose is to accumulate clinical data, obtain the outcome data of the intervention method and prove the feasibility of the study protocol. Forty-two patients will be selected as the sample size according to clinical experience.

### Statistical analysis

The results will be analysed by using the SPSS software (SPSS V.12.0 KO for Windows). The accepted level of significance will be p<0.05. Measurement data were expressed by mean number±SD, enumeration data expressed as a percentage.

The statistical analysis will be carried out based on the theory of intention-to-treat (ITT) analysis as well as per-protocol (PP) analysis. In the case of ITT analysis, missing data will be replaced according to the principle of the last observation carried forward and the maximum likelihood regression analysis. PP analysis will be conducted with patients who have received treatments >16 times and complete the case report form (CSF). χ^2^ Test is going to be performed for the situation of proportions; meanwhile, the analysis of independent sample t-tests is going to be conducted to examine the baseline discrepancies between the two groups. The significance of the differences in the various data in each group will be analysed with a paired t-test. On the basis of the baseline and temporary analgesic medicine dosage adjustment, continuous measurement results will be analysed using covariance test, and logistic regression analysis will be used for the two classification outcomes. Above two analyses will be present as difference in means or advantage ratio with 95% CIs.

### Ethics and dissemination

The protocol has been registered to ClinicalTrials.gov registry. Any revisions about the protocol will be documented in the ClinicalTrials.gov registry. Written informed consent will be obtained from all participants. The patients will be given adequate time to raise questions and to consider whether or not to involve in the study. We are going to publish the results of this trial in a peer-reviewed clinical journal to have widespread dissemination.

## Discussion

KOA is a common public health problem and a leading cause of disability. The results of this pilot study are going to concentrate on patients suffering from mild-to-moderate KOA and will investigate whether acupuncture can be a practicable and efficient therapy.

A suitable control group is critical for a well-designed clinical trial. On the basis of the literature review as well as clinical experiences, the acupoints in the MA group do not therapeutically affect KOA. Additionally, the dosage for acupuncture is sufficient. The protocol specifies the intervention of acupuncture to be a 20 min treatment which is applied 3 times weekly for 8 weeks, with 24 sessions in total permitted. Moreover, according to generality for the trial, the wide inclusion criteria will render it more possible that the participants fairly stand for those who have mild-to-moderate KOA. One potential limitation of this study is that acupuncturists are not blinded because of the nature of intervention. However, acupuncturists will not relate to the outcome assessments or data analyses.

The pilot trial will supply the clinical foundation as well as data that are demanded for evaluating the practicability for a large-scale RCT trial in the future.

### Trial status

This trial is currently recruiting participants.
